# Removal of Methylene Blue and Congo Red Using a Chitosan–Graphene Oxide-Electrosprayed Functionalized Polymeric Nanofiber Membrane

**DOI:** 10.3390/nano13081350

**Published:** 2023-04-12

**Authors:** Nethmi S. L. Dissanayake, Maadri A. Pathirana, Nandula D. Wanasekara, Boris Mahltig, Gayani K. Nandasiri

**Affiliations:** 1 Department of Textile and Apparel Engineering, Faculty of Engineering, University of Moratuwa, Moratuwa 10400, Sri Lanka; nethmidissanayake26@gmail.com (N.S.L.D.);; 2 Faculty of Textile and Clothing Technology, Hochschule Niederrhein-University of Applied Sciences, 41065 Mönchengladbach, Germany

**Keywords:** methylene blue removal, congo red removal, electrospinning, electrospraying, graphene oxide, chitosan, polyacrylonitrile

## Abstract

Untreated textile effluent may contain toxic organic pollutants that can have negative impacts on the ecosystem. Among the harmful chemicals present in dyeing wastewater, there are two frequently used organic dyes: methylene blue (cationic) and congo red (anionic). The current study presents investigations on a novel two-tier nanocomposite membrane, i.e., a top layer formed of electrosprayed chitosan–graphene oxide and a bottom layer consisting of an ethylene diamine functionalized polyacrylonitrile electrospun nanofiber for the simultaneous removal of the congo red and methylene blue dyes. The fabricated nanocomposite was characterized using FT-IR spectroscopy, scanning electron microscopy, UV-visible spectroscopy, and Drop Shape Analyzer. Isotherm modeling was used to determine the efficiency of dye adsorption for the electrosprayed nanocomposite membrane and the confirmed maximum adsorptive capacities of 182.5 mg/g for congo red and 219.3 mg/g for methylene blue, which fits with the Langmuir isotherm model, suggesting uniform single-layer adsorption. It was also discovered that the adsorbent preferred an acidic pH level for the removal of congo red and a basic pH level for the removal of methylene blue. The gained results can be a first step for the development of new wastewater cleaning techniques.

## 1. Introduction

The ecosystem and human health are both severely impacted by contaminated wastewater. It is suspected that organic dye pollutants in effluent streams from the textile, food, print, paper, and cosmetic industries pose health concerns to humans since those contain particles that are highly resistant to oxidization, biodegradation, photodegradation, and heat. Over 7 × 10^5^ tons of commercial dyes are produced annually, with the textile sector consuming two-thirds of that amount [1]. Additionally, it has been calculated that 10–20% of the dye produced is discharged into wastewater annually [2]. Numerous distinctive physical, chemical, and biological dye removal techniques have been documented in the literature, including membrane separation, chemical oxidation [3], coagulation [4], photodegradation [5], biodegradation [6], and adsorption [7,8,9]. Due to the low cost, ease of use, and environmental resistance, physical adsorption methods are highly utilized. Although chemical and biological methods for treating water have been considered, they are not considered practical solutions due to the fact that dye molecules have a higher resilience to biodegradation and oxidization.

Information is abundant on adsorbents designed to remove either cationic or anionic dyes. However, there is a lack of literature concerning the simultaneous removal of both cationic and anionic dyes. Although methylene blue (MB) is reported to be used widely to dye cotton and silk garments, it is a cationic dye that is non-biodegradable and hazardous, that is discovered in high levels in textile effluent streams [10]. Because of its affinity to the negative polar sites of water molecules, cationic dye dissociates into positive ions in an aqueous media [11]. Previous research has connected exposure to MB to serious health problems, including nausea, jaundice, hemoglobin M disease, excessive perspiration, stinging sensations, cyanosis, and quadriplegia [12,13,14]. CR is a benzene-based azoic anionic dye that is frequently utilized in the textile industry [15]. About 60–70% of the overall amount of dye used in the textile industry is composed of azo dyes, which are identified as organic compounds having one or more azo (N=N) groups [1]. Azo dyes are recognized as environmental hazards because they could produce poisonous aromatic amines when N=N linkages are cleaved by reductive processes [16]. Congo red (CR), a popular azo dye, undergoes reductive cleavage to yield benzidine, a recognized human mutagen, and carcinogen [17]. In addition to respiratory issues, eye and skin irritations are also linked with CR dye exposure [18]. Therefore, it is essential to remove harmful dyes such as CR and MB from the textile wastewater system.

Organic contaminant removal using nanomaterials as adsorbents has attracted research attention with the advancements in nanotechnology. Given the unique characteristics of electrospun polymeric nanofibers (EPNFs) such as the higher theoretical surface area, controlled pore sizes, and superior porosity, nanofibrous materials are being employed more and more in the field of wastewater treatment [19]. Electrospinning is a widely used technique for fabricating nanofibers of diameters varying from 50 nm to 1 μm [20]. In electrospinning, the morphology and size of the fibers could vary with the adjustment of the electrospinning parameters such as applied voltage, polymer concentration, collector-to-tip distance, and feeding flow rate. The fibers are formed through the elongation of a charged polymeric solution using an electric field, which overcomes the surface tension and elongates the polymeric jet into thin nanofibers towards a grounded collector. Technological and research advancements in the electrospinning technique’s ability to scale up have greatly increased its capability as a nanoscale adsorbent [21].

Polyacrylonitrile (PAN) is an extensively used polymer in manufacturing EPNFs, owing to its low density, and chemical and thermal stability [22]. Electrospun PAN nanofibers’ insufficient adsorption sites or lack of surface wettability limit their ability to remove dye pollutants from wastewater [22]. It has been suggested in the literature, that surface modification is the best method to overcome the limits in PAN’s ability to remove organic pollutants since it could impart functional groups to the PAN structure [23,24,25]. According to early research studies, introducing functional groups that include oxygen or nitrogen could considerably increase the adsorbing capacity of polymeric adsorbents. Amino functionalization is generally preferred due to its ability to form potent complexes with organic pollutants [23,26,27]. Conforming to the literature, Almasian et al. [23] were the first to create amino-functionalized electrospun PAN nanofibers to enable anionic dye removal. It has been shown that surface functionalization is essential for improving PAN EPNFs’ ability to adsorb materials. Mahmoodi et al. [28] developed a porous modified PAN nanofiber membrane by incorporating sodium carbonate, which also possesses a greater anionic dye adsorption capability. According to a study by Patel and Hota [29], the density of amino groups directly correlates with the adsorptive capacity of functionalized PAN EPNFs, which is modified using ethylenediamine (EN), demonstrating the greatest adsorption capacity for CR dye, which was reported to be 130 mg/g [29].

**Figure 1:**
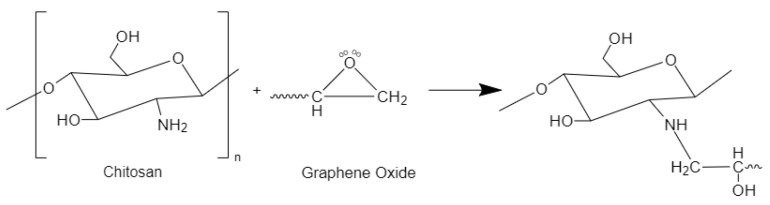
Cross-linking mechanism between chitosan and an epoxy group present on a graphene oxide surface.

The literature has reported several adsorbents, e.g., chitosan [30] and zeolite [31] as well as carbon-containing adsorbents such as activated carbon, carbon nanotubes, and graphene oxide for the removal of MB [32,33]. Due to their availability, intrinsic porosity, and ease of synthesis, carbon-based adsorbents are more frequently utilized than other materials capable of organic dye removal [34]. The literature highlights graphene oxide (GO) as a suitable carbon-based adsorbent for MB due to its 2D structure, high surface area, and the presence of functional groups that contain oxygen that have the potential to generate electrostatic interactions with positively charged cationic dyes. If GO is employed in its raw form, it can be difficult to recover GO from effluent streams as it creates stabilized colloidal particles that prevent the separation of phases. According to previous academic studies, adsorbents developed by crosslinking GO with various polymers might offer a feasible approach to overcome GO’s drawbacks as an adsorbent [35]. Chitosan (CS) is a biopolymeric material with abundant primary amino groups that has potential uses in the treatment of wastewater. According to Shao et al. [36], secondary amines can be formed when the epoxy groups are present in GO cross-linked with CS via the primary amino groups (Figure 1). Numerous research investigations have demonstrated the ability to encapsulate GO in a polymer matrix containing chitosan to absorb MB [37,38,39].

This study investigates the feasibility of a novel nanocomposite membrane for the simultaneous removal of two dyes, CR and MB, utilizing a CS–GO electrosprayed PAN–EN-electrospun membrane. The proposed nanocomposite structure consists of two layers, each designed with distinct chemical and structural properties for the optimal adsorption of MB and CR. The topmost layer is coated with a CS–GO electrospray, which is tailored to adsorb MB, while the lower layer, composed of a PAN–EN-electrospun membrane, is optimized for CR dye adsorption. This unique structure enables the removal of both cationic and anionic dyes. The EPNF membrane, tactically chosen as the bottom layer, exhibits a higher surface area, controllable pore structure, and regeneration capability. Additionally, its intrinsic affinity for organic dyes makes it an ideal candidate for removing CR. The designed nanocomposite membrane exhibits a high potential for removing both MB and CR, demonstrating promising results for future applications [19]. As supported in the previous literature, chitosan could firmly attach to a PAN-electrospun surface through the formation of hydrogen bonds [40,41,42]. The electrospray coating technique has been adopted to facilitate the deposition of the CS–GO solution onto the functionalized electrospun membrane, as it has certain advantages over conventional coating methods, e.g., enhanced wettability and adsorption capacity. Using electrospraying technology, the droplet size can be controlled, and as the diameters of the electrosprayed beads are known to be very small, a higher adsorptive surface area will be available on the fabricated nanocomposite membrane. The current research characterizes the designed adsorbent using scanning electron microscopy (SEM), Fourier transform infrared (FT-IR) spectroscopy, and drop shape analysis techniques, investigates the effect of process parameters, e.g., contact time, pH, and temperature, on MB and CR removal, and definitively ascertains the ultimate adsorptive capability of the solution, employing isotherm modeling to do so.

## 2. Materials and Methods

### 2.1. Materials

The GO was produced via powdered graphite acquired from Neochem International (Pvt) Ltd., Colombo, Sri Lanka, whereas the CS was created from shells of shrimps sourced from Homagama, Colombo, Sri Lanka. Polyacrylonitrile powder of a molecular weight of 150,000 was purchased from Shandong Natural Micron Pharm Tech Co., Ltd. in China. The chemicals used in the experiment, including ethylenediamine (EN, 99%), dimethylformamide (DMF, 99.8%), hydrochloric acid (HCl, 37%), sodium hydroxide pellets (NaOH, 97%), acetic acid (glacial, 100%), sulfuric acid (H_2_SO_4_, 99%), potassium permanganate (KMnO_4_, 99.9%), hydrogen peroxide (H_2_O_2_, 30%), ethanol (C_2_H_5_OH, 99%), and phosphoric acid (H_3_PO_4_, 85%) were obtained from Sigma Aldrich, Burlington, Massachusetts, United States and utilized without undergoing subsequent purification.

### 2.2. Extraction of Chitin and Deacetylation to Chitosan

The domestic shrimp shells gathered around Homagama were utilized as the main raw material for the extraction of CS. The shrimp shells were thoroughly washed with distilled water, carefully dried to eliminate excess water and ground to a fine powder. The chitin extraction process was completed in two sequential stages, i.e., (I) demineralization (1M HCl; *w*/*v*, 1:16; room temperature; 24 h), (II) deproteinization (2M NaOH; *w*/*v*, 1:14; room temperature; 48 h). Subsequently, the chitin was converted into chitosan through deacetylation (48% NaOH; *w*/*v*, 1:16; room temperature; 48 h), according to a standard chitosan synthesis technique reported by Hossain et al. [43] After each stage, the samples were neutralized using distilled water and dried thoroughly inside a vacuum oven at 80 °C.

### 2.3. Synthesis of GO

Utilizing graphite powder as a raw material and a modified Hummer*’*s method, the graphene oxide was synthesized [44]. The oxidization of graphite flakes was carried out for four hours in accordance with the synthesizing procedure described by Tissera et al. [45]. The graphite powder (1% wt) was added into a mixture of concentrated H_3_PO_4_:H_2_SO_4_ at a 1:9 ratio and KMnO_4_ (6% wt). The mixture was heated to 50 °C and stirred continuously for four hours using a magnetic stirrer. The initial dark purplish green color of the mixture changed to a dark brown hue during the reaction. To inhibit oxidation, the temperature of the mixture was reduced to room temperature by adding a mixture of 30% H_2_O_2_ and ice. At this stage, the mixture*’*s hue transformed to a dark yellow, suggesting a substantial level of oxidation in the graphite material [44]. It was washed three times with ethanol, once using 30% HCl and once with water after the remnant, solid material had settled. The solution was then centrifuged at 9000 rpm for an hour (Z 326 K, HERMILE, Gosheim, Germany), and the supernatant solution was then separated. The rinsing process was carried out until the pH level was between 4*–*5. The dehydration of the remaining solid material was carried out at 60 °C.

### 2.4. Preparation of Chitosan–Graphene Oxide Solution

A 2% chitosan solution was prepared by mixing chitosan powder with a 4% acetic acid solution and stirring the mixture continuously at 60 °C for 12 h. A solution containing 0.5% (*w*/*v*) of GO was obtained by dispersing GO in a 1% acetic acid solution. The obtained CS solution was transferred to the GO solution and stirred at 600 rpm for three hours. Ultrasound was applied for four hours in a bath sonicator to ensure the uniformity of the CS–GO solution.

### 2.5. Optimization of Electrospinning Parameters

PAN polymer solutions with different concentrations were created by mixing a dimethylformamide (DMF) solvent with PAN polymer powder. To obtain uniform polymer solutions, the PAN solutions were stirred using a magnetic stirrer for 12 h. The electrospinning process was conducted using an electrospinning instrument composed of a high-voltage unit and a feeding unit. For the optimization of PAN electrospinning parameters, a 21-gauge blunt needle attached to a 5 mL plastic syringe was loaded with the PAN solution. The feeding unit was attached to the syringe, and the feeding rate was set at 0.6 mL/hr. In this study, the PAN concentrations of 3 wt%, 6 wt%, 8 wt%, and 10 wt% were used. The applied voltages varied from 15–20 kV, and the needle tip-to-collector distances were 12 cm and 15 cm. A grounded metal plate covered using aluminum foil was utilized as the collector. When the needle was subjected to a high voltage, the solvent present in polymer droplets that were ejected from the needle tip became evaporated, and ultrafine PAN polymer fibers were deposited on the collector.

### 2.6. Fabrication of Polyacrylonitrile-Electrospun Membrane

Through the combination of the PAN powder with DMF and stirring the combination continuously over the course of 12 h, a 10 wt% PAN polymer solution was prepared. The produced 10 wt% PAN solution was inserted into a 5 mL syringe fitted with a metallic needle, which was then connected to a syringe pump set to dispense at a rate of 0.6 mL/hr. Subsequently, a high-voltage power source was utilized to apply a 20 kV potential to the positive terminal of the metallic needle tip, while the negative terminal was wired up with a collector covered in aluminum foil. The gap between the grounded metallic collector and the needle tip was adjusted to 15 cm, which allowed for the collection of ultrafine PAN polymer fibers on the collector surface.

### 2.7. Surface Functionalization of Electrospun PAN Membrane

**Figure 2:**
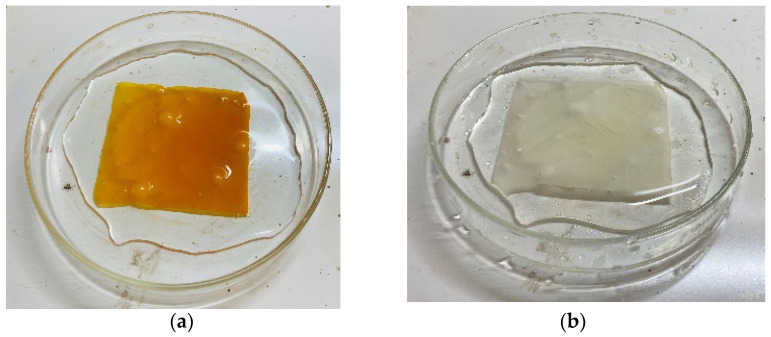
(**a**) Sodium hydroxide-modified polyacrylonitrile-electrospun nanofibrous membrane and (**b**) polyacrylonitrile-electrospun nanofibrous membrane functionalized with ethylenediamine.

The polyacrylonitrile nanofibrous membrane of a size of 6 cm × 5 cm was subjected to a 1 h immersion in 50 mL of a 15% NaOH solution at 60 °C [29], leading to a change in color from white to yellow (Figure 2a). Then, the fibrous membrane was rinsed using distilled water and dipped for 30 min at room temperature in 50 mL of a 1M HCl solution to neutralize the excess NaOH, restoring the white color. The results were in accordance with the research described by Patel and Hota [29]. The nitrile groups available on the surface of the hydrolyzed PAN nanofiber membrane were then replaced with amino groups through the treatment of a 10% EN solution (50 mL) for 5 h at ambient temperature. The modified electrospun membrane was then washed with distilled water following drying at ambient temperature on aluminum foil (Figure 2b).

### 2.8. Preparation of Ch–GO-Electrosprayed PAN–EDA Membrane

The prepared CS–GO homogenous mixture was inserted into a 10 mL metallic needle attached to a syringe. The syringe was connected to a pump specifically designed for syringes with a fixed feeding rate of 0.5 mL/h. The syringe pump, which had a constant feeding rate set at 0.5 milliliters per hour was utilized to feed the CS–GO solution. A high-voltage power supply of 20 kV was applied to the metallic needle tip. The aluminum foil attached to the PAN–EN functionalized membrane was used as the collector. The distance between the collector to the tip was set at 12 cm. Following electrospraying, the solvent was evaporated and the coated membrane was air-dried.

### 2.9. Characterization

For the determination of functional groups and chemical structures, FT-IR spectrums were taken on an FT-IR (FT-IR Bruker Vertex 80, Bruker Corporation, Billerica, MA, USA) within the 400 to 4000 cm^−1^ range. Graphene oxide, PAN, PAN–EN, and CS–GO electrosprayed PAN–EN membrane surface topography and morphology were examined using a SEM (Zeiss Evo 18 Research SEM, Carl Zeiss Microscopy, White Plains, NY, USA). The sample SEM micrographs were captured at a 10 kV accelerating voltage. Utilizing a UV-vis spectrometer, an abso+rbency evaluation of the dyes MB and CR on the produced membrane was performed (UV-3600, Shimadzu, Kyoto, Japan). To examine the surface wettability of the nanofibrous membranes, Drop Shape Analyzer (DSA100E, KRUSS, Hamburg, Germany) was utilized together with DSA25 and the ADVANCE software.

### 2.10. Adsorption Study of CS–GO Composite

In this study, the equilibrium adsorption capacity of the CS–GO-electrosprayed PAN–EN membrane for MB and CR dyes was evaluated in batch mode, using sealed sample bottles with a constant volume of adsorbent. The adsorption isotherm of MB was determined by varying the pH levels of the solution (pH 3, 5, 7, 9, and 11) and MB concentrations (ranging from 10 to 70 ppm) while maintaining a fixed adsorbent dosage of 5 mg. The fixed capacity of the MB solution in every specimen container was maintained at 25 mL. The experimental data obtained from the adsorption isotherm were analyzed to investigate the adsorption behavior of MB on the CS–GO-electrosprayed PAN–EN membrane. The solutions were prepared by diluting a 200 ppm stock solution of MB to the necessary concentrations. Using MB absorbance at varying concentrations, a linear calibration curve was developed following absorption measurements at a wavelength of λ = 664 nm. CR’s adsorption isotherm was achieved in batch mode under settings that included varied initial CR concentrations (10–70 ppm) and pH levels (pH = 3, 5, 6, 7, 8 and 9) with an adsorbent dose of 5 mg. Each sample bottle’s final CR solution volume was fixed at 25 mL. The solutions were created by diluting a 200 ppm stock solution of CR to the necessary concentrations. CR absorbance at a wavelength of λ = 497 nm with varying concentrations was utilized to develop a linear calibration curve. After 48 h, to reach the equilibrium state, the specimens were vacuum-separated from the mixture to remove the solid particles, and UV-vis spectroscopy (UV-vis 3600 spectrophotometer) was used to assess the absorbance of the residual dye solution. Both MB and CR’s equilibrium uptakes were determined using the following equation:

(1) $$Q_{e}=\frac{C_{0}-C_{e}}{W}\times v$$

In the given equation, *C*_0_ (mg/L) denotes the initial dye concentration, *C_e_* (mg/L) denotes the remaining dye concentration (MB or CR), Q_e_ (mg/g) signifies the amount of dye absorbed for every unit mass of the adsorbent while *W*(g) indicates the weight of the adsorbent material used, and *V*(l) represents the volume of the dye solution.

### 2.11. Adsorption Isotherm Study of CS–GO Composite

To determine the precise percentage of removal of the adsorbate, various isotherm models, Freundlich, Langmuir, and Dubinin–Radushkevich, were developed for describing the adsorption isotherm. The Langmuir isotherm model explains the equilibrium of a homogenous surface, in which the adsorbate is sorbed onto a monolayer. In contrast, the Freundlich isotherm model explains an adsorbent with a heterogeneous surface covered in multilayers of adsorbate molecules [46]. The mathematical expressions for the Langmuir model (2) and the Freundlich model (3) are provided.

(2) $$\frac{C_{e}}{q_{e}}=\frac{C_{e}}{q_{m}}+\frac{1}{K_{L}\cdot q_{m}}$$

(3) $$logq_{e}=\frac{1}{n}logC_{e}+logK_{F}$$

The equilibrium adsorption capacity is denoted by *q_e_* (mg/g), while *C_e_* (mg/L) represents the equilibrium concentration of the adsorbate solution. The Freundlich constants, n and *K_F_* (mg/g), are related to the intensity and adsorption capacity, respectively, whereas *q_m_* (mg/g) represents the maximal adsorptive capacity, and *K_L_* (l/mg) denotes the Langmuir adsorptive equilibrium constant [47]. By analyzing the results obtained from UV-vis experiments, the isotherm modeling studies for MB and CR were carried out by plotting graphs that conformed to Equations (1) and (3).

### 2.12. Reusability Study

To evaluate the adsorbent*’*s regeneration capacity, a 0.01 M NaOH solution was used to treat the dye-adsorbed CS–GO-electrosprayed PAN–EN membrane. The dye adsorbed on the nanocomposite was removed by stirring the NaOH treated membrane constantly at 150 rpm for 2 h. The adsorbent was washed in multiple instances with deionized water and dried in an oven to dry the membrane for 1 h at 60 °C. The nanocomposite membrane was subsequently reused to remove dyes in the next cycle. A reusability study was conducted to investigate the nanocomposite membrane*’*s regeneration capability of removing MB and CR dyes, with the regeneration capacity of the CS–GO electrosprayed PAN–EN membrane being studied over four consecutive cycles.

## 3. Results and Discussion

### 3.1. FT-IR Analysis

**Figure 3:**
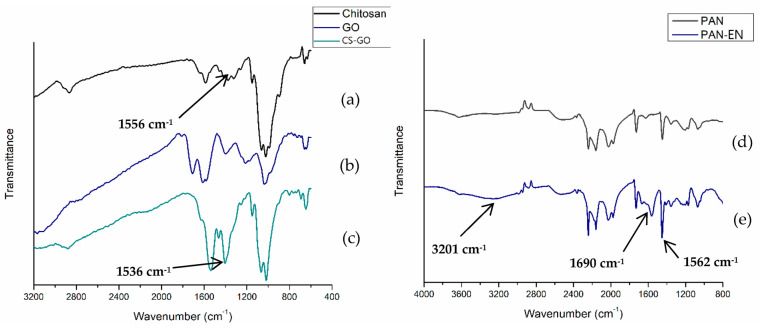
FT−IR spectra of (**a**) CS, (**b**) GO, (**c**) CS-GO membrane, (**d**) PAN and (**e**) PAN-EN electrospun nanofibers.

Figure 3a illustrates the FT-IR spectrum of the manufactured chitosan, which exhibits a prominent band in the frequency at a range of 3285 to 3356 cm*^−^*^1^, which corresponds to the O-H and N-H stretching vibrations. The presence of CH_3_ and CH_2_ groups in the biopolymer is indicated by a minor peak at 2864 cm*^−^*^1^, which corresponds to the stretching vibration of C–H bonds. The main amine and amide I bands respective to N-H bending are characterized by peaks at 1556 and 1642 cm*^−^*^1^, respectively. The peak observed at 1150 cm*^−^*^1^ in the FT-IR spectrum of CS suggests the presence of the C-O-C asymmetric stretching vibration, while the absorption peaks located at 1028 and 1065 cm*^−^*^1^ in the fingerprint region indicate the stretching vibrations of the C–O bonds. The absorption peaks correspond with findings by Queiroz et al. [48], Knidri et al. [49], Kumari and Rath [50], and Laaribi et al. [51].

Figure 3b of the FT-IR spectrum, which corresponds to produced GO sample, illustrates a significant peak at 3203 cm*^−^*^1^ that is assigned to O-H stretching vibrations. Aldehydes’ and ketones*’* C=O stretching vibrations correlate to the absorption band at 1709 cm*^−^*^1^. The carboxy stretching vibrations may be responsible for the modest absorptive peak at 1179 cm*^−^*^1^, while the peak in correspondence with the C-O-C stretching of the GO’s epoxy groups is determined at 1036 cm*^−^*^1^. These peaks match the results of Nazri et al. [52], Ciplak et al. [53], and Sabzevari et al. [37].

Figure 3c illustrates the FT-IR spectra of the produced CS–GO composite. The peak positions of chitosan and the composite appear to be noticeably similar. Peaks at C-H stretching (2872–2874 cm^−1^), C-O-C stretching (1066–1148 cm^−1^), and O-H and N-H stretching vibrations (3204–3249 cm^−1^) provide evidence of the mentioned equivalences. This could be elaborated by the chemical interaction (Figure 2) which takes place among the primary amino groups in CS and the epoxy groups present in GO, which results in the production of secondary amines by the primary amines and provides comparable FT-IR readings. A closer inspection of the FT-IR results reveals that the shift (blue) of N-H bend vibrations from 1556 cm^−1^ of CS to 1536 cm^−1^ of CS–GO, as described in the work by Shao et al. [36], indicating the reaction between epoxy and amine.

**Figure 4:**
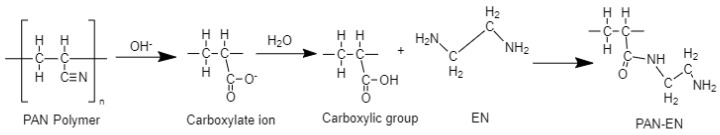
Functionalization of PAN membrane using ethylenediamine under alkaline conditions.

Figure 3d exhibits the FT-IR spectrum of PAN nanofibers and of the ethylenediamine (EN)-modified PAN membrane. The adsorption bands at 2513 and 1448 cm^−1^ in each spectrum correspond to the stretching and bending vibrations of MB groups, correspondingly. The noticeable peak at 2241 cm^−1^ in PAN represents the nitrile group (vibration related to the stretching of the triple bond between carbon and nitrogen (C≡N)). When PAN and sodium hydroxide were mixed with each other, the interaction between the nitrile group (C≡N) in PAN and sodium hydroxide (NaOH) generated carboxylate as a byproduct (Figure 4). In the PAN structure, the amino group of EN underwent a reaction with the carboxylate ions to produce an amide group when the PAN was treated with EN (Figure 4). The PAN–EN nanofibrous membrane exhibited adsorption bands in the vicinity of 2241 cm^−1^ due to the existence of unreacted C≡N. The common adsorption peaks at 3629 cm^−1^ could be used to represent the O-H groups of water molecules involved in hydrogen bonding. The presence of the adsorptive peaks at 3434, 1562, and 1690 cm^−1^ in PAN–EN, which are absent in the FT-IR spectra of untreated PAN nanofibers, and which correspond with the amines’ stretching vibrations, C=O stretching in the amide, and N-H bending vibration in the amide, respectively, indicates that the amide was formed. The findings resemble those of other research by Almasion et al. [23], Patel and Hota [29], and Jenab et al. [54].

### 3.2. Optimization of Electrospinning Parameters

The surface characteristics, i.e., the morphology and size, of the PAN-electrospun membranes fabricated with different electrospinning parameters were studied using a scanning electron microscope (SEM). To optimize the electrospinning parameters of PAN, the polymer concentration was varied, at 3 wt%, 6 wt%, 8 wt%, and 10 wt%. For the 3 wt% concentration, the sputtering of the PAN solution occurred and only droplets were formed instead of nanofibers. The formation of droplets may have resulted from an electric field distortion which contributed to the change in the profile of the surface of the liquid drop held at the end of the needle tip into a conical shape. The polymer solution’s low surface tension and inadequate molecular chain entanglements due to the low concentration allowed the electrically driven polymer jet to be raptured into droplets [55,56]. The nanofibers (NFs) fabricated using the 6 wt% and 8 wt% solutions formed beaded structures. With an increase in the concentration, the NFs’ shape changed from droplets to beaded NFs, which may have been caused by the increased molecular entanglement that prevented the jet from splitting. The beads formed by the 6 wt% solution were visible to the naked eye. The PAN NFs at the 8 wt% concentration were not uniform, and their beaded structures were visible under an optical microscope.

Table 1 shows the SEM images of the electrospun PAN membrane at various voltages, and the distance between the tip and collector with a constant 10 wt% polymer concentration and 0.6 mL/hr feeding rate. As shown in Table 1, the NFs fabricated with the 10 wt% solution were smooth and uniform. The concentration of the PAN solution was not further increased, because an increase in concentration reduces the stretching of the fiber jet which causes an increase in the diameter of NFs [57]. As shown in Table 1, the voltage was varied, keeping the PAN concentration (10 wt%), feeding rate (0.6 mL/h), and needle-to-collector distance (15 cm) constant. The resultant NF’s diameter varied from 395 nm to 265 nm. Under an increased voltage supply, a large electrostatic stretching force may have been exerted, causing the polymer jet to accelerate and stretch under the electric field. Higher stretching promotes the formation of NFs of lower average diameters [58]. Then, the tip-to-collector distance varied between 15 cm and 20 cm, keeping the other parameters constant. When comparing the results at 20 kV, the diameter of the nanofibers when the gap between the collector and the tip was 15 cm was 265 nm while when the gap between the collector and the tip was 12 cm, the diameter was 311 nm. The gap between the collector and the tip significantly influenced the strength of the electrostatic field, the duration of solvent evaporation, and the whipping route of the jets. Since beads would have been visible if the distance was too short or too long, it was demonstrated that a reasonable distance range was required to provide ample time for the PAN solution to stretch and evaporate the solvent prior to the deposition of NFs on the substrate [55,56]. The tip-to-collector distance of 15 cm formed uniform NFs of smaller diameters. Per the results obtained from the optimization study, the parameters of the 10 wt% PAN concentration, 15 cm tip-to-collector distance, 20 kV voltage, and 0.6 mL/hr feeding rate were used to fabricate the PAN-electrospun membrane for all the experiments outlined in the present study.

### 3.3. SEM Results

**Figure 5:**
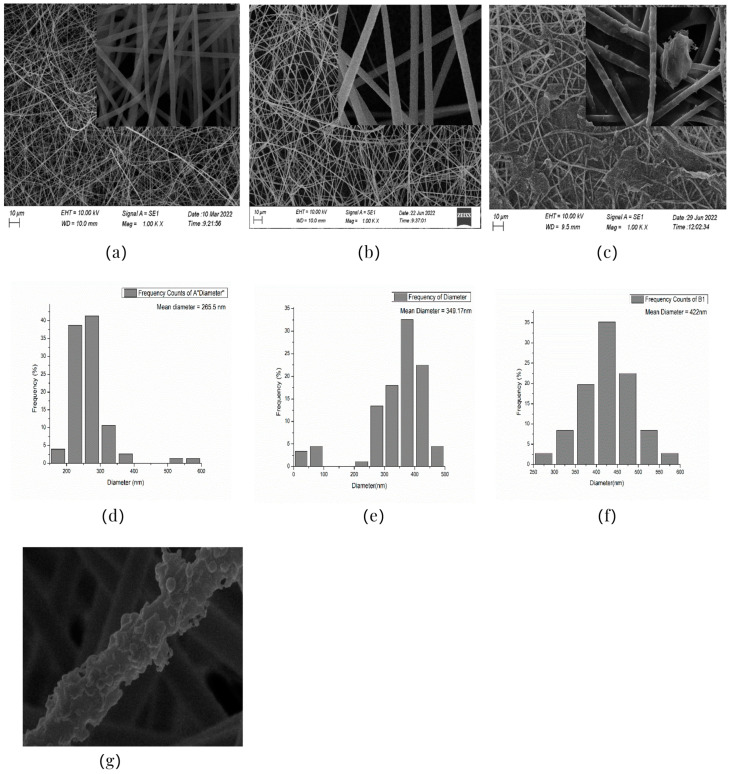
SEM images of (**a**) PAN membrane (**b**) PAN–EN membrane and (**c**) CS–GO-electrosprayed PAN–EN membrane; histograms of (**d**) PAN membrane and (**e**) PAN–EN membrane (**f**) CS–GO-electrosprayed PAN–EN membrane; SEM images of (**g**) magnified nanofiber of CS–GO-electrospun PAN–EN membrane.

A scanning electron microscope (SEM) was used for the examination of surface characteristics, i.e., the morphology and size, of the polyacrylonitrile-electrospun membrane (Figure 5a), PAN–EN membrane (Figure 5b), and CS–GO-electrosprayed PAN–EN-electrospun membrane (Figure 5c). Using the ImageJ application, fiber diameters were analyzed from SEM images, and histograms were generated. Histograms were used to calculate the mean diameter of each fiber sample. The histogram of the PAN fibrous mat is shown in Figure 5d, and the mean diameter of fibers was determined to be 266 nm, whereas the membrane comprised smooth, homogeneous nanofibers. The PAN fibrous membrane was modified with EN, and the mean diameter of fibers was 349 nm (Figure 5e). The functionalized PAN membrane appears to have had swollen nanofibers, but the smooth surface morphology of nanofibers remained unchanged. The EN-functionalized PAN membrane was electrosprayed with a chitosan–graphene oxide solution. Based on Figure 4f, the mean diameter of fibers was calculated to be 422 nm. The increase in the PAN nanofiber diameters may have been due to the expansion of PAN nanofibers subsequent to the EN surface modification and due to the layered thickness of the CS–GO coating. The PAN membrane’s even surface seems to have transformed into one that was rougher following electrospraying. The agglomeration which appears in the SEM images of the electrosprayed nanocomposite (Figure 5c) membranes may have been caused by the aggregation of GO particles. In order to ensure the uniform dispersion of GO in chitosan, the CS–GO solution was subjected to bath sonication. However, there was a considerable time lapse between ultrasonication and surface coating via electrospraying due to the logistics involved in electrospraying, i.e., the time taken to remove the sonicated sample from the bath sonicator to the electrospinning unit and the preparatory procedure involved in electrospraying. This can be the reason why an agglomerated particle is seen in the SEM images of the electrosprayed membrane. Figure 5g illustrates the magnified SEM image of a CS–GO-electrosprayed PAN–EN nanofiber, which shows the roughness created by the CS–GO coating on the PAN–EN membrane.

### 3.4. Contact Angle Measurements

**Figure 6:**
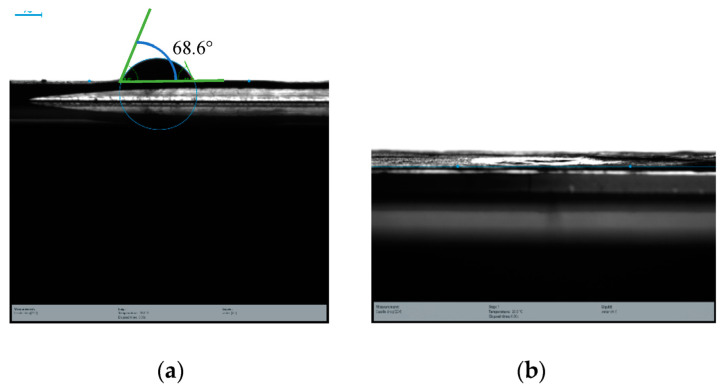
Static contact angle of (**a**) PAN membrane and (**b**) CS–GO-electrosprayed PAN–EN membrane.

Owing to its excellent chemical, mechanical, and thermal characteristics, PAN is a polymer that is frequently employed to produce EPNFs; however, due to its poor surface wettability, dye absorption is hindered. Surface modification has been suggested in the literature as a method for enhancing the hydrophilicity of polymeric nanofibers. Surface coating and wet chemistry are two post-treatment methods used in the current study for the manufactured PAN EPNF. While the former refers to the surface coating of the functionalized PAN membrane by electrospraying, the latter relates to the modification of PAN with EN by the chemical interaction of the amino-functional units in EN with the nitrile groups in PAN. As illustrated in Figure 6a, a static contact angle of 68.6° was measured from the PAN EPNF using the sessile drop method. Following the surface alterations, the fabricated EPNF showed perfect wettability, as shown in Figure 6b, but the static contact angle of the sample could not be measured because the water droplet was instantly absorbed into the sample. According to previous studies, an EPNF*’*s hydrophilicity relies on its homogeneity, polarity, and layered thickness [22]. An effective coating of the top tier is responsible for a noticeable rise in the surface wettability of the CS–GO-electrosprayed PAN–EN-electrospun nanofiber membranes. The study*’*s observations are coherent with the results described by Lou et al. [40]. It is possible that the polarity that GO imparted had a role in the increase in hydrophilicity. Thus, with the use of analytical reasoning, it could be inferred that the dye adsorption capacity might be enhanced by the improved surface wettability of the membrane.

### 3.5. Adsorption Study

Linear calibration curves for MB and CR absorbance at varied concentrations were produced using the absorption measurements of MB and CR performed at wavelengths of λ = 664 nm and λ = 497 nm, respectively.

#### 3.5.1. Effect of Solution pH on Adsorption

To investigate the impact of pH on the adsorptive capacity of a CS–GO-electrosprayed PAN–EN-electrospun membrane for MB, a starting concentration of 5 ppm MB was arranged, and the pH of the solution was ranged to be from 3 to 10 via incremental additions of 0.1M HCl/0.1 M NaOH solutions. This was carried out because the ionization of GO is affected by the pH of the solution due to the presence of carboxylate functional groups on GO, which experience partial separation in water-based solutions to generate hydrogen and carboxylate ions. By introducing hydroxide (OH^−^) ions, the equilibrium would shift towards the ionization of carboxylate groups, according to Le Chatelier’s principle.

**Figure 7:**
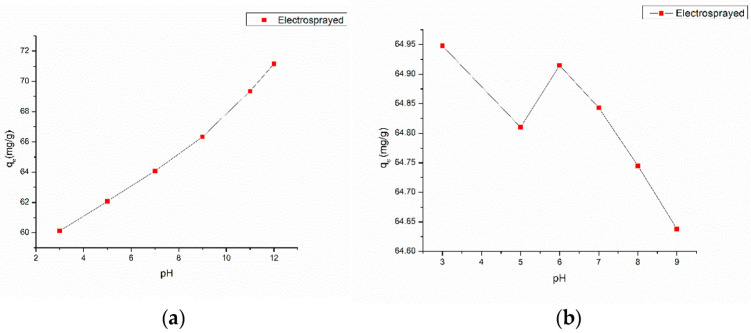
Absorption capacity of (**a**) MB and (**b**) CR as a function of pH at contact time = 48 h, adsorbent dosage = 5 mg, and temperature = 300 K.

**Figure 8:**
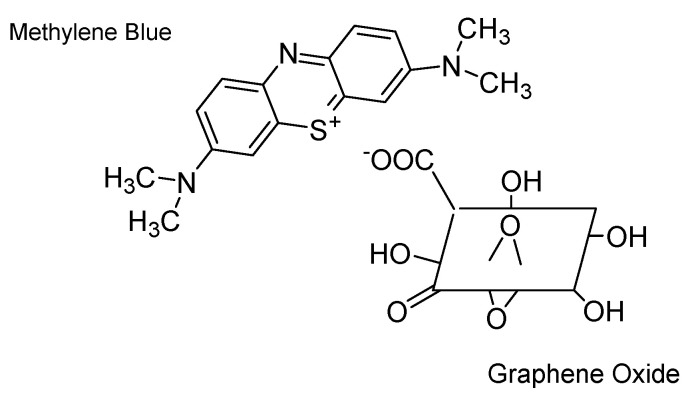
Electrostatic interaction between MB and GO.

The observed increase in the adsorption capacity of methylene blue (MB) with increasing pH levels, as shown in Figure 7a, can be associated with the increase in the ionization of carboxylic acid groups on the surface of graphene oxide (GO). This phenomenon would lead to the increased attraction of cationic MB molecules to the GO surface, as depicted in Figure 8. Conversely, the amino groups on the polyacrylonitrile (PAN) and chitosan components of the adsorbent would undergo protonation at lower pH levels, resulting in the production of NH_3_^+^ ions. In the solution, these ions would in turn reject the cationic dyes, leading to undesired outcomes. Therefore, it can be inferred that a high basic pH environment is preferable for the produced sorbent to effectively remove MB from the solution. The pH trends observed in our study are consistent with those reported in previous studies by Huang et al. [59] and Yang et al. [60]. This indicates that the influence of pH on the adsorption behavior of cationic dyes is a widely acknowledged phenomenon in the scientific community. 

**Figure 9:**
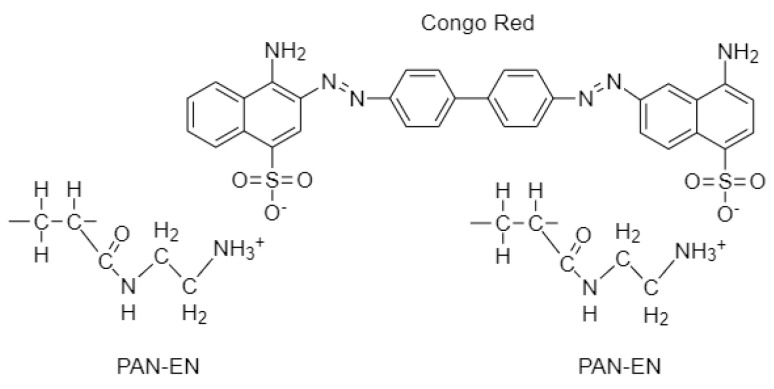
Electrostatic interaction between CR and PAN-EN membrane.

The protonation or deprotonation of amino functional groups on CS and PAN–EN is controlled by the solution pH. A CR solution that has a starting concentration of 20 ppm and an adsorbent dose of 10 mg/25 mL was utilized to evaluate how the adsorption capacity for CR of the electrospun membrane manufactured was affected by changes in pH. The solution*’*s pH was varied from 3 to 10 using 0.1M NaOH and 0.1M HCl solutions. The adsorption capability of EPNF peaked at pH 3, varied up to pH 6, and then steadily decreased when the pH value was raised, as shown in Figure 7b. Low pH levels caused the amino functional groups on PAN–EN (Figure 9) and CS to protonate, increasing the static electric interaction between the adsorbent and the anionic dye. On the other hand, the negative charge on the functionalized surface increased when the amino group deprotonated at increasing pH levels. Electrostatic repulsion caused a substantial drop in the percentage of overall dye elimination [61]. Despite the fact that CR*’*s peak adsorptive capacity was recorded at pH 3, the dye*’*s color changed from red to blue at this pH. According to Nadjia et al. [62], the maximum peak for CR in the UV-visible spectrum does not have a significant change in the pH range of 6–10, which is in accordance with the current work. Therefore, pH 6 was considered to be the optimum pH for CR removal. The best adsorption capacity was demonstrated for MB at higher pH levels, followed by CR at pH 6. However, following the final stage of washing during the coloration process in the textile industry, the effluent has a pH of 7*–*8 [63]. A neutral pH of 7 was chosen for the adsorption isotherm studies after taking all of these aspects into consideration.

#### 3.5.2. Effect of Contact Time

**Figure 10:**
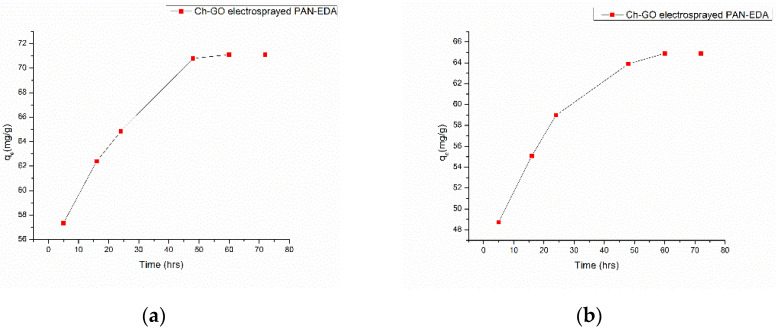
The absorption capacity for (**a**) MB and (**b**) CR as a function of contact time at temperature = 300 K, pH = 7, and adsorbent dosage = 5 mg.

An adsorbent and a dye require an adequate contacting duration to achieve equilibrium. According to the data displayed in the graphs in Figure 10a,b, a period of 48 h was needed to attain equilibrium. Additionally, by changing the temperature, the relationship between temperature and the adsorption capacity for the dyes MB and CR on the chitosan-graphene oxide-electrosprayed PAN-EN membrane was examined. However, there was no remarkable alteration in the concentration in which equilibrium was noticed.

### 3.6. Adsorption Isotherm

Table 2 displays the optimum parameters for the two models that were inferred from the two isotherm fittings. Table 3 presents the isotherm graphs according to the Langmuir and Freundlich models for MB and CR. Table 4 compare the optimum adsorptive capacity (q_max_) of adsorbents cited in the literature that are intended to eliminate CR and MB. According to the higher correlation coefficient of the Langmuir model for MB dye, the removal of MB using the nanocomposite mat highly matches that under the Langmuir isotherm model, which is suggestive of uniform and single-layer adsorption. The CS–GO-electrosprayed PAN–EN-electrospun membrane*’*s experimentally determined q_max_ is 219.3 mg/g, which is slightly lower than the q_max_ of the GO powder (243 mg/g) and much greater than that of the raw chitosan (11 mg/g) [30,33].

Due to the epoxy–amino reaction between GO and chitosan, there was a decrease in functional epoxy sites of the GO, which resulted in a minor reduction in the manufactured nanocomposite membrane*’*s adsorption capacity for MB compared to that of the graphene oxide. However, when compared with other nanomaterials based on carbon such as carbon nanotubes (CNTs) [64] and graphene [65], the fabricated nanocomposite membrane had a higher adsorption capacity. This occurred because graphene oxide has functional groups that contain oxygen, dispersed over a significant contact area, increasing the adsorption capability. Additionally, it can be shown that the fabricated adsorbents*’* q_max_ exceeded the q_max_ of the other CS–GO composites mentioned in other studies [38,39,66]. According to the literature, the removal of organic dyes from a solution utilizing pure, mixed, or modified electrospun membranes occurs due to the electrostatic attractions, van der Waal forces, π-π stacking, hydrogen bridge bonds, and pore-filling [67,68,69,70,71]. PAN has a maximum adsorptive capacity of 42.67 mg/g for MB, according to Haider et al. [72]. This is because the presence of nitrile groups allows PAN to create hydrogen bonds with positively charged dyes [72]. In this research, EN was used to modify a PAN-electrospun membrane, causing the nitrile groups in PAN to react chemically with EN (Figure 6). With methylene blue, the unreacted nitrile groups may have generated hydrogen bonds, as mentioned as part of the FT-IR analysis. Furthermore, a negatively charged intermediate carboxylate ion was created during the interaction between NaOH and PAN. The cationic dye molecules may have formed electrostatic attraction forces with the carboxylate ions that were available on the surface of the PAN. The increased adsorptive capacity of the membrane studied under this research for MB compared to that of the other CS–GO composites documented in the literature can be explained by the fact that the q_max_ of the developed EPNFs for MB exceeded that of other PAN-functionalized EPNF membranes fabricated in other studies [73].

Due to the greater correlation coefficient, the removal of CR from the CS–GO-electrosprayed PAN–EN membrane similarly fitted that under the Langmuir isotherm model, and the q_max_ was measured by means of experimentation to be 182.43 mg/g. Due to the presence of electrostatic attraction forces between the negatively charged CR and the protonated amino groups, the nanocomposite membrane was capable of removing the CR dye. The data presented in Table 4 leads to the conclusion that the fabricated EPNFs*’* q_max_ for the removal of CR is greater than the q_max_ of the PAN–EN membrane stated by Patel and Hota [29]. This could be explained by the availability of a coating on top made from chitosan, which provided more anionic dye adsorption due to the protonated amino sites in chitosan. Chitosan is a biopolymer that is a promising and affordable dye removal adsorbent known for its non-toxicity and biodegradability; however, it has a low surface area in its raw form, which restricts its inherent adsorption capability [74]. The current research study shows the capability to enhance the adsorptive capacity of the PAN membrane by coating a significant portion of the surface with a chitosan–graphene-oxide layer. The manufactured EPNFs outperformed other adsorbents described in the research studies, as stated in Table 4, in terms of their adsorption capabilities. Therefore, it could be concluded that the CS–GO-electrosprayed PAN–EN membrane offers an efficient approach for removing MB and CR from aqueous solutions.

**Table 4:** Comparison of several adsorbents’ adsorption capacities for MB and CR.

Absorbent	Dye Removed	Adsorption Capacity (mg/g)	Sources
Carbon nanotubes	MB	46.2	[64]
Graphene	MB	153.9	[65]
Magnetic graphene oxide/β-cyclodextrin	MB	93.97	[66]
SrAl_2_O_3._Bi^3+^/graphene	MB	42.92	[75]
Graphene oxide–cyclodextrin–chitosan–ferric oxide	MB	84	[39]
Graphene oxide–chitosan–ferric oxide	MB	95	[38]
Graphene oxide–ferric oxide	MB	167	[76]
Graphene oxide/cobalt oxide nanocomposite	MB	40	[77]
Hydrogels loaded with magnetic reduced graphene oxide	MB	119	[78]
Raw chitosan	MB	11	[30]
Powdered graphene oxide	MB	243	[33]
Polyacrylonitrile nanofibers	MB	42.67	[13]
Oxime-grafted polyacrylonitrile	MB	102.15	[73]
Zinc-augmented polyacrylonitrile nanofibers	CR	25.64	[79]
PET fibers grafted with 4-VP	CR	17.3	[80]
Functionalized polyvinyl chloride–graphene-polyaniline fibers	CR	40.0	[81]
Zinc oxide/Tin oxide porous nanofibers	CR	90.8	[82]
Silicon dioxide–bohemite core/sheath fibers	CR	24.3	[83]
Chitosan hydrobeads	CR	92.59	[84]
Commercial activated carbon	CR	66.67	[85]
Acticated carbon loaded with palladium nanoparticles	CR	76.9	[86]
Acticated carbon loaded with gold nanoparticles	CR	66.7	[86]
Polyacrylonitrile–ethylene diamnine-functionalized membrane	CR	130	[29]
Chitosan–graphene oxide-dipcoated PAN–EDA nanocomposite	MB	201	[87]
Chitosan–graphene oxide-dipcoated PAN–EDA nanocomposite	CR	152	[87]
CS–GO-electrosprayed PAN–EN functionalized membrane	MB	219.3	Present work
CS–GO-electrosprayed PAN–EN functionalized membrane	CR	151.745	Present work

### 3.7. Reusability Study

**Figure 11:**
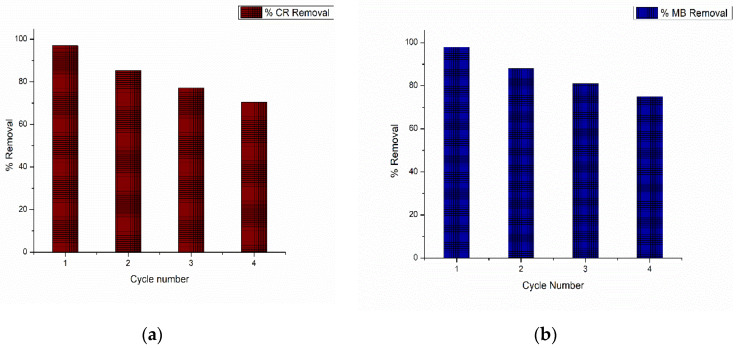
The percentage of elimination of dyes (**a**) CR and (**b**) MB as a function of cycle number using chitosan–graphene oxide-electrosprayed PAN–EN membrane.

In four sequential cycles, the adsorption of the CR and MB dyes by the regenerated chitosan–graphene oxide-electrosprayed nanocomposite membrane was investigated and the results for the two composites are depicted in Figure 11a,b. It was shown that within the first four cycles, the percentage of removal for CR was 70.4% and that for MB was 75.1%. Thus, it could be concluded that a CS–GO-electrosprayed PAN–EN membrane could be utilized as an effective adsorbent for CR and MB dye decontamination with adequate reusability, which makes it a promising adsorbent in terms of wastewater treatment.

## 4. Conclusions

A novel adsorbent, the CS–GO-electrosprayed EPNF membrane was successfully developed for the elimination of the CR and MB dyes. PAN-electrospun membranes were fabricated with different electrospinning parameters, and an optimized set of variables was determined for the designed structures. It was concluded that the fibers of the lowest diameters were generated using a 10 wt% PAN concentration with a 0.6 mL/h feeding rate, keeping a 15 cm gap between the collector and tip and a voltage of 20 kV. The average fiber diameter for the optimized structure was determined to be 266 nm. The SEM images further revealed that the fibers were swollen subsequent to EN-functionalized surface modification for which 349.2 nm was calculated as the average diameter. However, the smooth morphology of the untreated PAN nanofiber was unaffected. According to the CS–GO-electrosprayed electrospun nanofiber SEM image, it was concluded that the smooth surface was converted to a relatively rough exterior with the average diameters falling in the average of 422 nm. Additionally, it was observed that in the nanocomposite membrane with an electrosprayed CS–GO top tier, GO was agglomerated. Owing to the CS–GO tier’s successful coating, contact angle measurements from the sessile drop technique demonstrated that the PAN membrane modified with EN had good wettability. The fabricated adsorbent’s responsiveness to contact time, temperature, and solution pH to the removal of MB and CR was investigated, and the findings listed below were established; as a result of the carboxyl groups on GO becoming ionized, the MB adsorption capacity appears to have been more beneficial in a high pH solution whereas for CR removal it was favorable in an acidic medium due to amino protonation. The adsorption process for both dyes did not face a significant influence from the temperature, and both dyes reached the equilibrium after 48 h. The effectiveness of the dye adsorption was quantified via isotherm modeling, which confirmed a maximal capacity for absorption of 182.48 mg/g for CR and that of 219.3 mg/g for MB associated with the electrosprayed nanocomposite membrane, which showed monolayer homogeneous adsorption and was successfully fitted with the Langmuir adsorption isotherm model. It was further revealed that the adsorbent favored a basic pH level for MB removal and an acidic pH level for CR removal. The novel CS–GO-electrosprayed PAN–EN nanofiber membrane designed and fabricated in this study to assist in the removal of CR and MB was demonstrated to be efficient and to surpass many other adsorbents identified in prior research.

## 5. Future Perspectives

Future advances seem to be plausible with the proposed adsorbent described in the present work. Future research studies could aim to improve the durability of the nanocomposite membrane by enhancing its mechanical strength, regeneration, and reusability, which could ultimately contribute to its upscaling potential. The thickness of a membrane is directly proportional to its mechanical strength; therefore, electrospinning for a longer duration or stacking multiple EPNFs on top of each other could increase the mechanical strength of the nanocomposite membrane. The mechanical characteristics of EPNFs should thus be the subject of further investigation. Modified EPNFs are generally anticipated to be a significant area of research with significant future potential.

## Figures and Tables

**Table 1:** Optimization of electrospinning parameters for PAN-electrospun membrane.

Conc. (%)	Voltage (kV)	Flow Rate (mL/h)	Tip-to-Collector Distance (cm)	Diameter (nm)	SEM Image
10	10	0.6	15	395	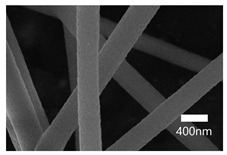
10	12	0.6	15	362	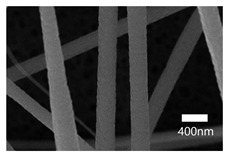
10	15	0.6	15	315	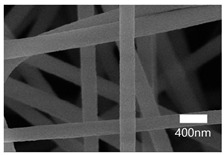
10	18	0.6	15	288	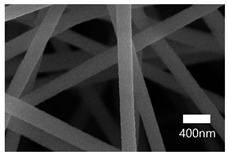
10	20	0.6	15	265	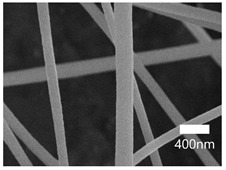
10	15	0.6	12	397	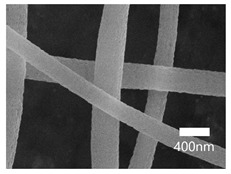
10	18	0.6	12	321	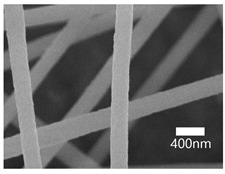
10	20	0.6	12	311	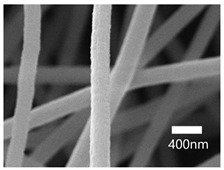

**Table 2:** Outcomes of Langmuir isotherm and Freundlich isotherm models.

	Langmuir Isotherm			Freundlich Isotherm		
Dye	q_max_ (mg/g)	K_L_(L/mg)	R^2^	K_F_	n	R^2^
MB	219.3	0.277879	0.99846	74.31	3.5880	0.94099
CR	182.48	0.000253	0.99915	21.16	1.5299	0.98317

**Table 3:** Adsorption isotherm graphs of Langmuir and Freundlich isotherm models for the dyes MB and CR at temperature = 300 K, pH = 7, adsorbent dosage = 5 mg, and contact time = 48 h.

Model	Methylene Blue	Congo Red
Langmuir model	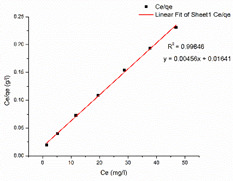	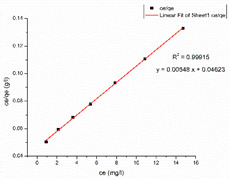
R^2^	0.99846	0.99915
Freundlich model	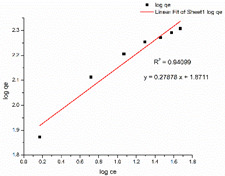	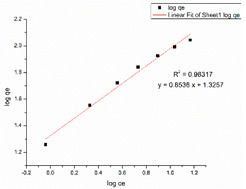
R^2^	0.94099	0.98317

## Data Availability

Data available on request.
